# Endovascular Treatment of Intracranial Aneurysms in Small Peripheral Vessel Segments—Efficacy and Intermediate Follow-Up Results of Flow Diversion With the Silk Vista Baby Low-Profile Flow Diverter

**DOI:** 10.3389/fneur.2021.671915

**Published:** 2021-05-28

**Authors:** Marie-Sophie Schüngel, Ulf Quäschling, Erik Weber, Manuel Florian Struck, Jens Maybaum, Nikolaos Bailis, Felix Arlt, Cindy Richter, Karl-Titus Hoffmann, Cordula Scherlach, Stefan Schob

**Affiliations:** ^1^Institute of Neuroradiology, University Hospital Leipzig, Leipzig, Germany; ^2^Department of Anaesthesiology, University Hospital Leipzig, Leipzig, Germany; ^3^Department of Neurosurgery, University Hospital Leipzig, Leipzig, Germany; ^4^Department of Neuroradiology, Clinic & Policlinic of Radiology, University Hospital Halle, Halle (Saale), Germany

**Keywords:** flow diversion, low-profile flow diverter, silk vista baby, small cerebral vessels, cerebral aneursym

## Abstract

**Background and Purpose:** Low-profile flow diverter stents (FDS) quite recently amended peripheral segments as targets for hemodynamic aneurysm treatment; however, reports on outcomes, especially later than 3 months, are scarce. This study therefore reports our experience with the novel silk vista baby (SVB) FDS and respective outcomes after 8 and 11 months with special respect to specific adverse events.

**Materials and Methods:** Forty-four patients (mean age, 53 years) harboring 47 aneurysms treated with the SVB between June 2018 and December 2019 were included in our study. Clinical, procedural, and angiographic data were collected. Follow-ups were performed on average after 3, 8, and 11 months, respectively. Treatment effect was assessed using the O'Kelly Marotta (OKM) grading system.

**Results:** Overall, angiographic follow-ups were available for 41 patients/45 aneurysms. Occlusion or significant reduction in aneurysmal perfusion (OKM: D1, B1–B3 and A2–A3) was observed in 98% of all aneurysms after 8 months. Only 2% of the treated aneurysms remained morphologically unaltered and without an apparent change in perfusion (OKM A1). Adverse events in the early post-interventional course occurred in seven patients; out of them, mRS-relevant morbidity at 90 days related to FDS treatment was observable in two patients. One death occurred in the context of severe SAH related to an acutely ruptured dissecting aneurysm of the vertebral artery.

**Conclusion:** The SVB achieves sufficient occlusion rates of intracranial aneurysms originating from peripheral segments, which are comparable to prior established conventional FDS with acceptably low complication rates. However, alteration of a hemodynamic equilibrium in distal localizations requires special attention to prevent ischemic events.

## Introduction

Endovascular treatment of intracranial aneurysms has experienced significant improvements in recent years (1). Most importantly, the introduction of flow diversion has driven the strategy away from intra-aneurysmal manipulation toward stepwise reconstruction of the affected segment. The comparatively novel technique allows one to circumnavigate the probation of the fragile aneurysm sac, which bears the risk for procedural rupture—and hence, fatal outcome in about a third of these cases (2). By implanting the densely woven mesh into the parent vessel, the aneurysm neck is covered and blood flow is directed away from the aneurysm orifice. Subsequently, thrombus is formed in the aneurysm sac and a novel layer of endothelium grows along the scaffold of the device (3).

Initially, flow diverter stents (FDS) were indicated for aneurysms arising from the petrous to the clinoid segment of the internal carotid artery (4). After this technique has been securely established and convincing outcomes in otherwise untreatable cases (5), flow diverters were used for smaller branches of the anterior intracranial and even the posterior circulation (6, 7). Accompanying the success of the technique, low-profile flow diverters, for example, the Silk Vista Baby (SVB, Balt, France), the p48MW (Phenox, Germany), and the FRED Junior (Mircovention, USA), have been developed and are now applied with increasing frequency to small and peripherally located, aneurysm-harboring segments of the intracranial arteries (8).

Few studies on the safety and feasibility of these FDS are available; however, reports on intermediate or long-term outcomes after treatment with low-profile FDS, especially concerning peripheral segments of the cerebral vessels, are lacking (9). Our institution has participated on the pre-market release of the SVB and reported first experience with the device (10). However, only early follow-up results were available. Therefore, our presented study aims to report the intermediate outcomes of patients treated with the Silk Vista Baby (SVB) low-profile FDS in our neurovascular center.

## Materials and Methods

### Ethics Approval

The institutional ethics committee approved our retrospective analysis of a prospectively maintained database including cases between June 2018 and October 2020 (local IRB no AZ 208-15-0010062015). Informed consent was waived from the IRB regarding the scientific use of anonymized clinical data.

### Study Design

The study comprises patients suffering from unruptured and ruptured aneurysms as well as clinically manifesting segmental arterial disease (for example, dissecting aneurysms) of the internal carotid artery terminus, anterior cerebral artery complex, middle cerebral artery, and vertebral and basilar artery who were treated with the SVB. Unruptured aneurysms were treated primarily with the SVB or had undergone endovascular pre-treatment (coiling or flow diversion) and exhibited significant relapse after initial treatment. Decision for endovascular therapy was made after discussion of each case in the interdisciplinary cerebrovascular board, consisting of neurosurgeons, neuroradiologists, and neurologists.

Demographic data, localization, size, and morphology of each aneurysm as well as procedural and post-procedural adverse events in combination with angiographic follow-ups were collected for analysis. Table 1 provides an overview of our patient database.

**Table 1:** Demographic data and follow-up results.

**Patient**	**Location**	**Previous SAH**	**Neck width (mm)**	**Dome width (mm)**	**Dome height (mm)**	**Parent artery diameter (mm)**	**Treatment strategy**	**Device dimensions**	**OKM after FD**	**OKM 1st FU (mean FU after 2.6 ‘months)**	**OKM 2nd FU (mean FU after 7.7 months)**	**OKM 3rd FU (mean FU after 10.6 months)**
1	A1/2–left	Fisher IV^*1^	2.2	3	2.3	2	Primary	SVB 2.25 × 15	A1	D1	D1	n.a.
2	M2–right	no	2	5.6	6.6	2	Primary	SVB 2.25 × 15	C3	D1	D1	n.a.
			3	3.8	3				A1	D1	D1	
3	A1/2–left	Fisher IV	3.8	5.5	7.3	2.5	Plug and pipe	SVB 2.25 × 1520	A1	D1	D1	n.a.
4	A2/3–right	no	1.2	1.7	2.2	1.8	Primary	SVB 2.25 × 1510	A1	D1	n.a.	n.a.
5	A1/2–left	no	2	4.5	4.3	2.2	Primary	SVB 2.25 × 1510	A2	D1	n.a.	n.a.
								SVB 2.25 × 15				
6	A1/2–right	Fisher IV	2.5	3.6	5.5	2	Plug and pipe	SVB 2.25 × 15	A1	D1	D1	n.a.
7	PICA left	Fisher IV	2.3	3.2	5	2.5	Plug and pipe	SVB 2.25 × 10	A1	D1	D1	n.a.
8	AcomA^*2^	No	2	3.4	4.1	1.9	Primary	3 × SVB 2.25 × 15	A3	C2	n.a.	n.a.
9	A1/2–right	Fisher IV	2	4	5	2.5	Plug and pipe	SVB 2.25 × 15	A1	D1	D1	D1
10	C6–right	no	4.5	5.8	8.6	3	Primary	SVB 3.25 × 20	A2	D1	D1	n.a.
11	RCP–right	Fisher IV^*1^	3.8	5.4	5.3	3.3	Primary	SVB 3.25 × 25	A1	A1	A3^*3^	A2
12	PICA–left	Fisher IV	2	5.3	5.8	2.8	Plug and pipe	SVB 3.25 × 10	A1	B1	B1	n.a.
13	A1/2–right	Fisher IV	2.5	4	5	2.5	Plug and pipe	SVB 2.25 × 15	A1	D1	D1	n.a.
14	A1/2–left	Fisher IV	2	3.3	3.9	2.1	Plug and pipe	SVB 2.25 ×15	A1	D1	n.a.	n.a.
15	RCP–left	Fisher IV^*1^	4.1	4.6	3	3.5	Primary	SVB 3.25 × 20	A1	B1	D1	n.a.
								SVB 3.25 × 25				
								1 WEB				
16	A2/3 right	no	1.8	2.9	3.2	1.8	Plug and pipe	SVB 2.25 × 10	A1	D1	n.a.	n.a.
17	C7 – left	Fisher	2.8	2.2	4	3.2	Plug and	SVB 3.25 × 20	A1	C1	C2	n.a.
	RCP–left	IV	1.9	1.7	2		pipe		A3	A3	A3	
18	M1–left	Fisher IV	2	4.6	5.1	3	Plug and pipe	SVB 3.25 × 20	A1	B1	D1	D1
19	A1/2–left	no	2	2.5	3.5	2	Primary	SVB 2.25 × 15	A1	B2	B2	B2
20	C6–left	Fisher IV	2.4	3	5.3	3.5	Plug and pipe	SVB 3.25 × 25	A1	D1	D1	n.a.
21	PICA–left	Fisher IV	3.9	3.9	8.3	2.6	Plug and pipe	SVB 2.75 × 25	A3	D1	D1^*4^	n.a.
22	M1–right	no	3.5	6	6	2.6	Plug and pipe	SVB 2.25 × 15	A1	A1	A1	n.a.
								SVB 2.75 × 15				
23	A1/2–left	Fisher IV	4.9	5.9	4.8	2.6	Plug and pipe	SVB 2.75 × 20	A1	B1	B1	B1
24	A1/2–right	Fisher IV	3.9	5.3	3.9	2.2	Plug and pipe	SVB 2.25 × 20	A1	D1^*5^	D1	n.a.
25	A1/2–right	Fisher IV	2.2	3.3	3.3	2.2	Plug and pipe	SVB 2.25 × 15	A2	D1	D1	D1
26	A1/2–left	no	2.5	2.8	2.9	1.8	Primary	SVB 2.25 × 15	B1	D1^*5^ ^*6^	n.a.	n.a.
27	V4/PICA–left	no	2.8	6.0	7.0	3.0	Primary	SVB 2.75 × 15	A3	B3	C2	n.a.
28	M1–right	Fisher IV^*1^	2.3	2.6	3.7	2.2	Primary	SVB 2.25 × 15	A1	A1	D1	D1
29	RCA	Fisher IV	8	14	12	2.6	Plug and pipe	SVB 2.75 × 20	A2	A2	D1^*5^	D1^*5^
30	A1/2–left	No	1.7	2.1	1.7	1.7	Primary	SVB 2.25 × 15	A2	n.a.	n.a.	n.a.
31	A1/2–right	Fisher IV	3.2	3.4	3.4	2.2	Plug and pipe	SVB 2.25 × 15 SVB 2.25 × 20	A1	C2	n.a.	n.a.
32	BA	No	–	–	–	2.5	Primary	2 × SVB 2.75 × 15	–	–	–	–
33	A1/2–right	Fisher IV	2.7	2.6	2.9	2.4	Revision	SVB 2.25 × 20	A2	D1 (right) C3 (left)	n.a.	n.a.
34	M1–left M1/2–left	Fisher III	3 5.8	3.2 10	3.1 18	2	Primary Plug and pipe	SVB 2.25 × 20	A2 A3	A2 D1	A2 D1	A2 D1
35	BA	No	1.6	1.6	2	1.8	Primary	SVB 2.25 × 15 Rebel 2.5 × 8	A1 Reconst.	n.a.	n.a.	n.a.
36	V4–right	Fisher III	–	–	–	3.1	Primary	SVB 2.75 × 15 SVB 2.75 × 20 4 x p48_HPC 3 × 18 (4) 3 x p48_HPC 3 × 15 (3) P48_HPC 3 × 12 Rebel 4.5 x 12	A1	A2	n.a.	n.a.
37	C6–right	No	6	7.1	5.1	3.5	Plug and pipe	SVB 2.75 x 15 SVB 2.75 x 20 SVB 3.25 x 20 SVB 3.25 x 25 3 x p48_HPC 3 x 18 ilk 5 x 40	A2	A2	n.a.	n.a.
38	C6–left	No	4	7	9	3.2	Primary	SVB 3.25 x 25	A3	D1	D1	D1
39	C6–left	No	5.4 3.6	7 3	7.5 3.4	3.1	Primary	SVB 3.25 x 25	A3 A2	C1 D1	n.a.	n.a.
40	A2/3–left	No	3.6	5	5	2	Plug and pipe	SVB 2.25 x 15	A1	A2	B2	n.a.
41	C6/7–right	Fisher IV	1.9	8.3	6	3.1	Plug and pipe/ revision	SVB 3.25 x 15	A3	B2	n.a.	n.a.
42	A2/3–left	No	1.8	2.3	2.6	1.9	Primary	SVB 2.25 x 10	B2	D1	n.a.	n.a.
43	A1/2–left	Fisher IV	2	3	2.4	2.7	Plug and pipe	SVB 2.75 x 15	A1	D1	n.a.	n.a.
44	M1/2–left	No	5	9	14	2.3	Primary	SVB 2.75 x 20 2 Coils	A3	B2	C2	n.a.

*1 *Patient priorily suffered SAH due to aneurysm of different location*.

*2 *Both right and left A1-A2 junctions were treated via flow diversion as the AcomA aneurysm got influx from both A1 segments (Double-C-stenting)*.

*3 *Patient underwent aneurysm retreatment with PED as the initially implanted SVB had contracted*.

*4 *Angiographic FU after 5months revealed asymptomatic occlusion of the parent artery (left V4)*.

*5 *FU shows sufficient flow diversion of the A1-A2 junction, however, angiography revealed contralateral aneurysm influx*.

*6 *Patient underwent retreatment and flow diversion of the contralateral A1-A2 junction with further SVB (look at patient 33)*.

### Antiplatelet Regimen and Endovascular Treatment

In the elective setting, dual antiplatelet therapy (DAPT) was initiated 24 h prior to treatment. The loading dose consisted of 500 mg of acetylic salicylic acid (ASA) and 180 mg of Ticagrelor. DAPT was then continued for 12 months with 100 mg of ASA and 180 mg of Ticagrelor given daily; the latter was administered in two doses of 90 mg every 12 h. In one case, DAPT was performed with 100 mg of ASA and 75 mg of Clopidogrel, as the patient already had been on DAPT for cardiac indication. Mono anti-platelet therapy with 200 mg of ASA twice a day (SAPT) was performed in one case of acute subarachnoid hemorrhage, related to a ruptured fusiform-dissecting vertebra-basilar aneurysm.

All interventions were performed under general anesthesia. Prior the procedure, a bolus of 5000 international units of heparin was given via the introducer sheath prior catheterization of the supra-aortic vessels. For the endovascular procedure, tri-axial access was established via the right common femoral artery using an 8F introducer sheath, a 6F guiding catheter (Neuron Max, Penumbra, Alameda, California, USA), and a 6F distal access catheter (Sofia, Microvention, Aliso Viejo, California, USA). In proximal locations (ICA, M1, V4, and BA) the Headway 17 (Microvention, Aliso Viejo, California, USA) was used for SVB implantation. In distal segments, mostly in the anterior cerebral artery segments, the Excelsior SL10 (Stryker Neurovascular, California, USA) was used for implantation of the smaller SVB models (2.75 mm and 2.25 mm diameter), as reported previously.

Sufficient opening of the implanted device and patency of the parent artery were controlled immediately after flow diverter implantation and again 15 min later. Also, potentially delayed perfusion of covered branches was controlled angiographically after deployment. In two cases, a covered branch exhibited significantly delayed perfusion and required further pharmaceutical intervention. For this, a bolus of Eptifibatid (©Integrilin, GlaxoSmithKline, Ireland) was given initially (180 μg/kg) and was continued as infusion therapy for 24 h (0.2 μg/kg per min). In both cases, no further treatment was necessary.

### Post-interventional Course

Electively treated patients were extubated directly after the procedure and were monitored continuously at our intensive care unit (ICU) for at least 24 h. Emergency patients were extubated in the further course at the ICU depending on their neurologic condition. Moreover, non-enhanced cranial computed tomography (CCT) and standardized neurologic examination were performed for every patient within 48 h post procedure in order to detect or exclude potential haemorrhagic or ischemic complications.

### Follow-Up Regimen

Efficacy of flow diversion was assessed immediately after flow diverter deployment using the O'Kelly-Marotta (OKM) grading scale (11). Efficacy of the treatment was re-assessed angiographically aiming for follow-up DSAs 3, 9, and 24 months after implantation and compared to the initial OKM grading.

## Results

### Patient Population and Aneurysm Characteristics

Intermediate follow-up results from patients treated with the low-profile SVB flow diverter were available in 44 individuals (31 female and 13 male patients with a mean age of 52.6 years ranging from 18 years to 83 years) in our analysis. Those 44 patients harbored a total of 47 aneurysms and were treated between June 2018 and December 2019 in our institution.

The majority−41 cases—were suffering from saccular intracranial aneurysms. The remaining three patients suffered from dysplastic segmental, partially stenosive basilar artery disease and presented as acute stroke in our emergency department.

**Figure 1:**
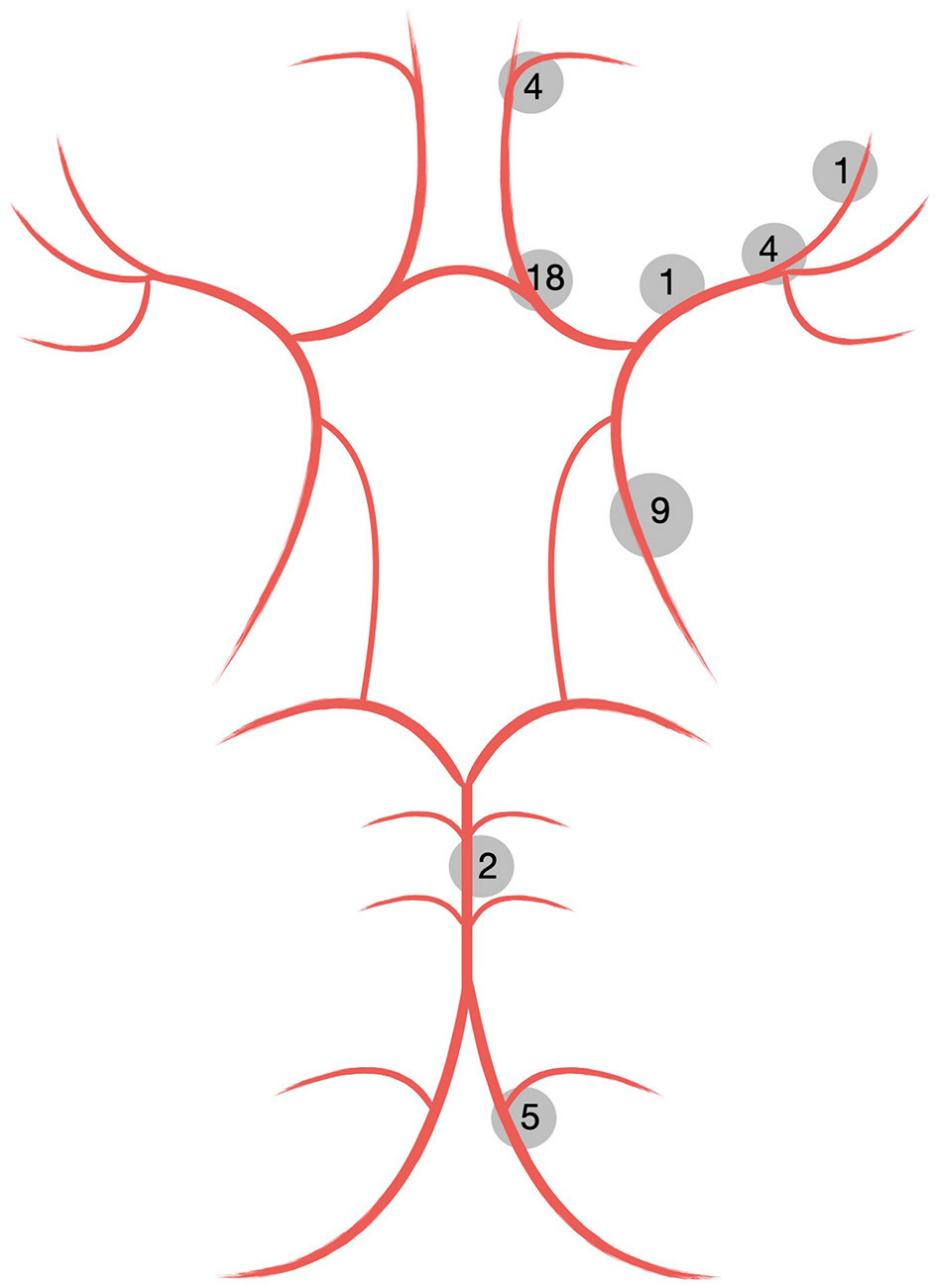
Distribution of cerebral aneurysms treated with the SVB.

Figure 1 graphically summarizes the anatomical distribution and corresponding frequencies of all included aneurysms. Table 1 provides an overview of the treated patients and the corresponding lesions.

### Treatments

Overall, 44 patients were treated with the SVB. Of those, 21 were primarily treated with flow diversion, 22 underwent retreatment after preceding coil embolization, and 1 patient underwent revision of an unsuccessful case of flow diversion with insufficient aneurysm occlusion in follow-up imaging.

In 36 cases, a single SVB was implanted, and in 6 patients, two overlapping SVB were used for sufficient coverage of the target lesion. One patient required implantation of three SVB (two unilaterally, one contralaterally) due to a wide-necked aneurysm of the anterior communicating artery (AcomA) with significant additional contralateral perfusion.

Four patients required implantation of additional devices to achieve technically sufficient results. The first patient suffered from an aneurysm originating from the left-hand M1-M2 segment that measured 9 × 14 mm and revealed signs of substantial mural inflammation, i.e., contrast enhancement of the thickened aneurysm wall together with peri-aneurysmal edema. To promote immediate stasis and relief of transmural force, two coils were loosely implanted in jailing technique as reported earlier (12).

In three patients, further stents were implanted synergistically. In the first of those cases, four SVB were implanted to treat an aneurysm located in a segment with large-caliber differences and a highly challenging, short distal landing zone. Primary attempt was to implant a first-generation Silk+ flow diverter (Balt Extrusion, Montmorency, France), which had failed due to insufficient definition of the distal landing zone with significant subsequent retraction.

Another patient was treated with a balloon-mounted coronary stent (REBEL, Boston Scientific, Maple Grove, USA) to reconstruct a high-grade stenosis proximal to the aneurysm-bearing segment. The remaining patient was treated with an additional low-profile FDS (p48MW_HPC, Phenox, Bochum, Germany) to treat extensive, long-segmental alterations with large differences in the proximal and distal landing zones.

### Technical and Clinical Adverse Events

#### Material-Related Adverse Events

In four patients, the SVB shortened immediately after insertion. In two of those cases, undersizing was decisive in retrospect. In the remaining two patients, the tortuosity of the target vessel was the underlying cause for malplacement requiring additional implantation of a second SVB in telescoping (stent-in-stent) technique.

In a fifth patient, the second follow-up after 5 months revealed distal device shortening resulting in insufficient aneurysm coverage. Related to significant differences in size of the proximal and distal landing zone, which were causative for shortening, retreatment was performed using an appropriately sized Pipeline 2 Shield (Medtronic, Covidien, USA).

All cases of shortening exclusively occurred during the first months after introduction of the SVB related to the rationale of implanting as little foreign material into the target vessel as possible.

#### Peri-Interventional Adverse Events

Two patients experienced peri-interventional branch occlusion or significantly delayed perfusion in the downstream territory. In one of the patients, the distal left-hand side ACA territory showed delayed perfusion after implantation of the SVB. To avoid stroke and permanent disability, body weight adapted intravenous application of Eptifibatid (GlaxoSmithKline, Ireland) was started immediately. The treatment significantly improved perfusion of the ACA territory in the angio suite. However, after waking up from general anesthesia, the patient exhibited a right-hand side hemiparesis and aphasia. Immediate cranial computed tomography revealed focal hypodensity in the left-hand side cortical MCA territory as an early sign of infarction, causative for the neurological deficit. Opacification of the MCA territory had been unremarkable during and at the end of the procedure. We therefore attribute the infarction to be a consequence of the long duration of the technically challenging intervention (ca. 4 h) together with comparatively low blood pressure during the procedure. At the last follow-up, 10 months after flow diversion, only mild speech disturbances remained.

The second patient experienced an asymptomatic transient occlusion of a temporal MCA branch, which was successfully treated with Eptifibatid as described above.

#### Adverse Events During the Early Post-interventional Period

Delayed adverse events occurred in seven patients.

**Figure 2:**
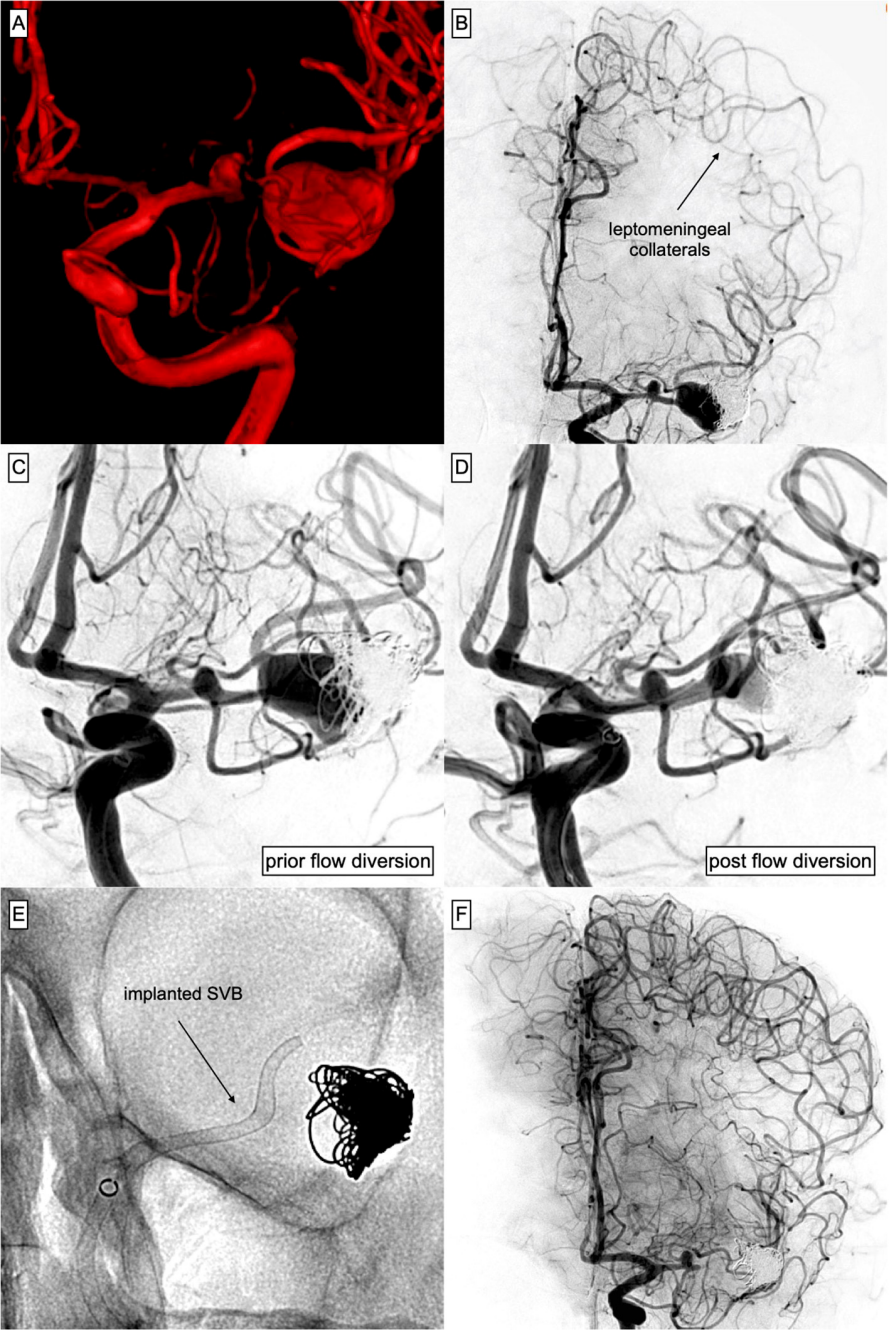
Complex course of a ruptured aneurysm arising from the MCA-bifurcation, treated with Plug & Pipe. **(A)** 3D reconstruction of the acutely ruptured, complex MCA-bifurcation aneurysm measuring 10 mm × 18 mm. **(B)** DSA 3 weeks after protective coiling of the ruptured aneurysm aiming for fundus protection and preservation of the bifurcation. Note the distinct ACA–MCA collaterals that had developed with SAH-associated subacute vasospasm. **(C)** Aneurysm after coiling, prior TO SVB implantation. Also note the coincidental aneurysm arising from the M1 segment and the stenosis preceding the aneurysmal orifice. **(D)** Control injection after implantation of the SVB [**(E)** shows the corresponding radiograph] into the MCA. The device covers the proximal M1 aneurysm and extends into the superior branch of the bifurcation, significantly reducing aneurysmal inflow. **(E)** Non-enhanced radiograph corresponding to **(D)**. **(F)** Three days after SVB implantation, the patient had developed a subtle right-sided facial paresis and fluctuating aphasia. Immediate DSA revealed no opacification of the distal MCA including the bifurcation-aneurysm but patency of the proximal SVB including the coincidental M1 aneurysm and a temporal MCA branch arising from the aneurysm base. However, ACA–MCA collaterals completely supplied the peripheral MCA territory distal to the occluded segment. A significant perfusion gradient between the proximal M1 segment and the stenotic pre-bifurcation segment had developed, which culminated in the manifestation of a distinct watershed zone after SVB implantation. Blood pressure was raised and the neurological deficit ceased subsequently. The patient had recovered completely after 48 h.

Permanent stent occlusion with transient neurologic deficits was observed in one individual. The patient had suffered from acute aneurysmal SAH caused by one of two aneurysms originating from the left-sided MCA bifurcation and developed delayed ischemia due to classic SAH-associated vasospasm. He was initially treated with endovascular coiling; however, retreatment was required related to aneurysm relapse. After the patient had recovered completely, SVB was implanted for definitive treatment of both aneurysms. Three days post procedure, the patient presented with fluctuating aphasia and subtle facial paresis. Re-angiography revealed absent opacification of the distal, SVB-covered M1–M2 segments with sufficient antegrade perfusion of the proximal M1, as well as SVB-covered segment and its side branches. Overall, no territorial or segmental perfusion deficit was apparent, as compensatory leptomeningeal collaterals originating from the ipsilateral ACA, which had developed during subacute vasospasm after SAH, were supplying the distal portion of the M1–M2 segments retrogradely. Figure 2 provides a detailed illustration of the case. At the last follow-up, 8 months after flow diversion, the patient revealed subtle residual speech disturbance.

Transient stent occlusion occurred in one patient after FDS implantation for treatment of an ICA aneurysm originating from the orifice of the left posterior communicating artery. Five h post procedure, the patient suddenly presented with a right-sided hemiparesis. Re-angiography revealed in-stent thrombosis resulting in distinctly reduced perfusion of the downstream ICA territory. After administration of Eptifibatid (GlaxoSmithKline, Ireland, 180 μg/kg) the angiogram revealed complete resolution of the thrombus and improved perfusion. No further treatment was required and the patient's neurologic deficits resolved completely.

Acute infarction in the aftermath of flow diversion appeared in one patient, who had initially suffered from aneurysmal SAH, which was treated with coiling. The broad-based AcomA aneurysm relapsed and required an additional intervention. Flow diversion in crossover technique was considered to be the only sufficient option. SVB was implanted from the right A2 segment into the left A1 segment. After implantation of the flow diverter, control injection revealed a delayed opacification of the left ACA territory. Eptifibatid was given as described above and improved perfusion of the depending ACA territory. However, the patient presented with reduced vigilance during the postinterventional course and developed partial bilateral cortical infarction in the ACA and MCA territory.

Two patients suffered from hemodynamically relevant vasospasm manifesting between 1 and 3 weeks after endovascular treatment, a phenomenon that we reported earlier (13).

**Figure 3:**
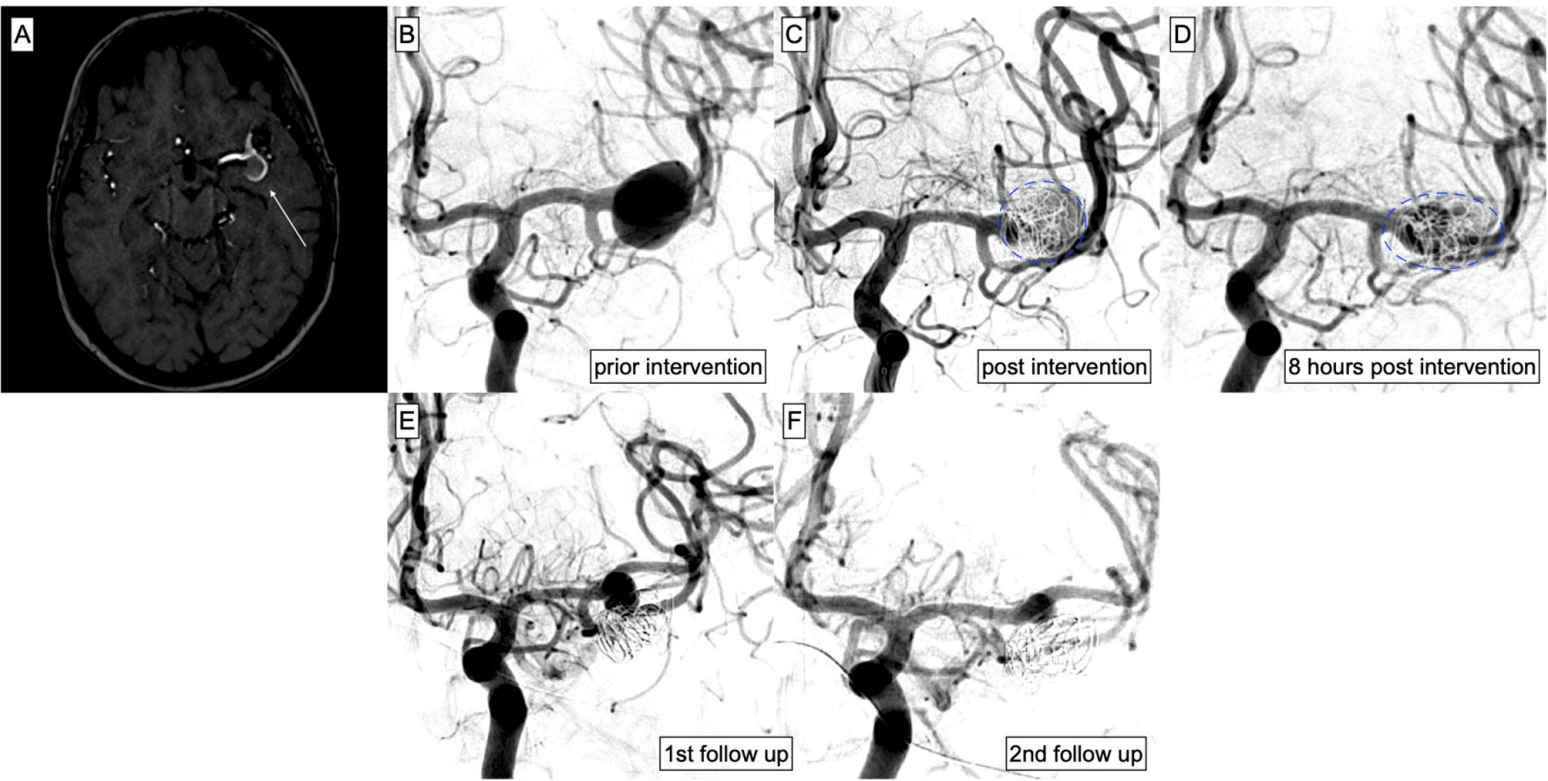
Endovascular treatment of an MCA aneurysm on the left side. MRI was performed due to fluctuating aphasia and hemiparesis. **(A)** Time of flight angiography revealed the aneurysm (white arrow) located at the left MCA bifurcation measuring 15 mm × 14 mm × 11 mm. **(B)** DSA confirmed the broad-based aneurysm. The patient refused open surgery and decided for endovascular treatment. **(C)** The aneurysm had grown 2 mm in size and its morphology had changed within 24 h, justifying again the prompt treatment by indicating a highly unstable situation. Therefore, decision was made for immediate protective coiling and flow diversion. The SVB was successfully implanted after loose coiling of the aneurysm sac (proximal landing zone: M1, distal landing zone: superior branch of the MCA bifurcation). **(D)** Eight h post intervention, the patient developed increasing hemiparesis of the left side. DSA was performed to exclude potential stent occlusion. The vessel proved patent; however, the morphology of the aneurysm and the coil package had changed again (highlighted in blue), indicating inflammatory changes of the aneurysm. Anti-inflammatory medication was given and the patient's symptoms resolved completely within 5 days. **(E)** The first regular follow-up 3 months post-treatment revealed a stable situation and decrease of the perfused aneurysm part/lumen. **(F)** One month later, DSA was performed again to decide on the further course of anti-inflammatory therapy. The perfused part of the aneurysm had further decreased and anti-inflammatory medication was discontinued.

In one case, MRI revealed extensive wall enhancement of a large MCA bifurcation aneurysm together with substantial peri-aneurysmal edema. In this patient, transient worsening of the pre-existing brachial paresis occurred 6 h after SVB implantation related to the progressive inflammatory mass effect of the aneurysm. Prophylactic anti-inflammatory medication (Dexamethasone 4 mg every 8 h) had already been given and was then amended by additional Celecoxib 100 mg daily. Neurologic deficits resolved completely after a period of 5 days. Figure 3 provides an overview of the respective case.

One patient died in the aftermath of extensive endovascular treatment of an acutely ruptured, multi-segmental dissecting vertebra-basilar aneurysm causing major SAH due to episodes of uncontrollable intracranial pressure.

### Early and Intermediate Aneurysm Occlusion Rates

Overall, angiographic follow-ups were available for 41 patients, harboring a total of 45 aneurysms. The occlusion rates after approximately 3, 8, and 11 months, were evaluated according to the O'Kelly-Marotta scale.

#### Early Follow-Up Results (Mean of 2.6 Months)

Early angiographic follow-ups revealed subtotal or complete aneurysm occlusion (OKM C1–C3 and D1) in 28 aneurysms (62%) and significant reduction of the residually perfused portion of the aneurysm sac (OKM B1-B3) in eight further aneurysms (18%).

In summary, the first follow-up indicates early sufficiency of the treatment in approximately 80% of the cases.

In one case, implantation of a singular SVB in the A1–A2 segment for treatment of a predominantly unilaterally filled AcomA aneurysm resulted in an angiographically sufficient result post implantation (OKM D1). However, after establishing a novel hemodynamic equilibrium after 4 months, the aneurysm was re-perfused from the contralateral A1 segment and required retreatment with a second contralaterally placed SVB.

Prolonged aneurysm opacification with delayed washout (OKM A2–A3) was achieved in six aneurysms (13%). Only three aneurysms (7%) did not show early apparent changes in perfusion after implantation (OKM A1).

#### Intermediate Follow-Up Results (Mean of 7.7 and 10.6 Months)

The third follow-up did not reveal relevant changes compared to the second follow-up. Therefore, results are presented together. At both time points, 34 aneurysms (76%) revealed subtotal or complete occlusion (OKM: D1 and C1–C3).

**Figure 4:**
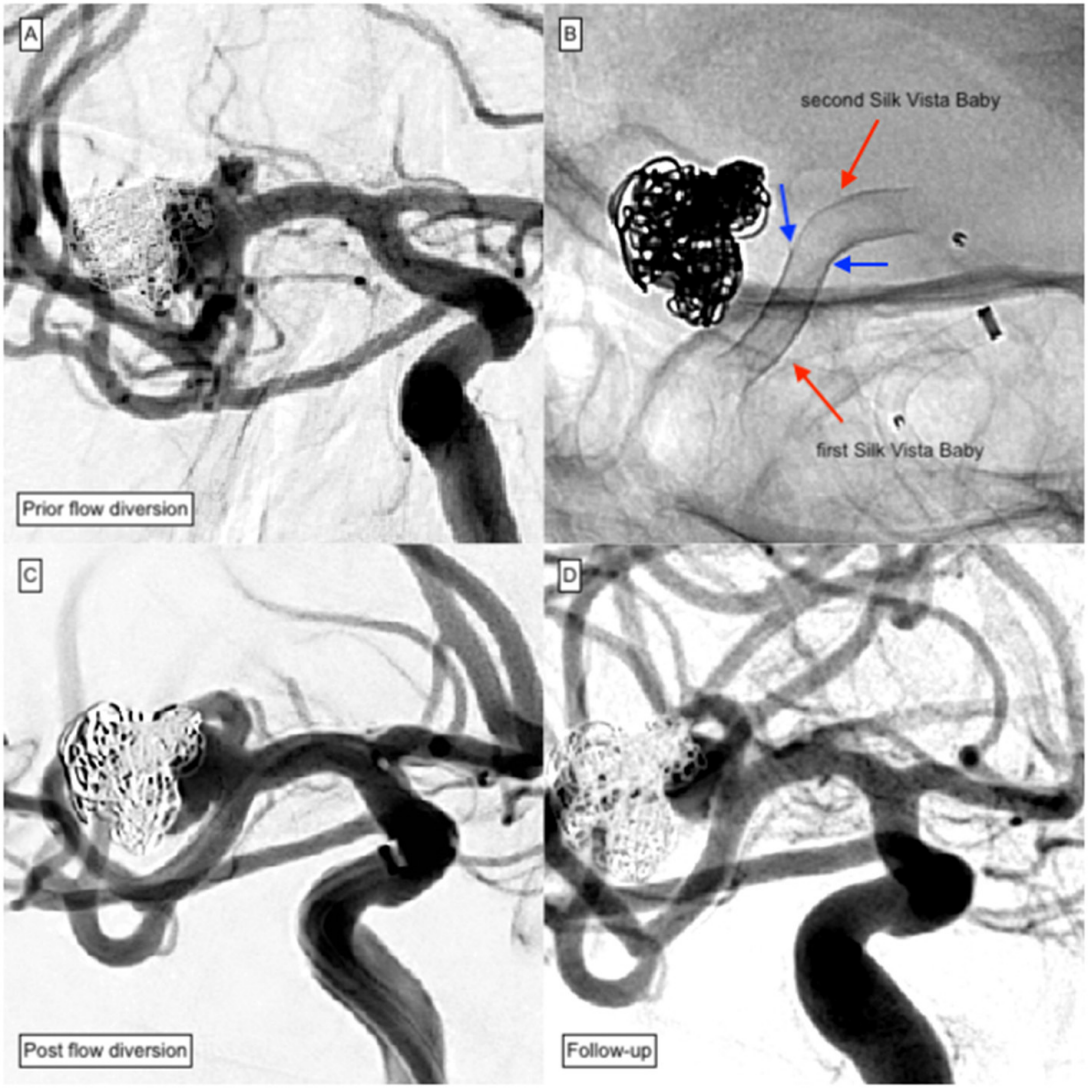
Plug and Pipe treatment of a previously ruptured MCA aneurysm of the right-handed side. Due to the difference in diameter between the M1 and M2 segments, extensive proximal shortening occurred after implantation of the first SVB and required complementary implantation of a second SVB. **(A)** DSA of a non-ruptured saccular aneurysm located at the right-handed side MCA bifurcation after protective coiling shows significant reperfusion and coil compaction. Decision was made for retreatment with SVB. **(B)** The first implanted SVB had shortened proximally (blue arrows) and did not sufficiently cover the aneurysm. Consequently, implantation of an additional device in telescoping technique was performed. **(C)** The control injection showed the timely opacification of all MCA branches including the covered superior truncus. **(D)** To avoid ischemic complications, DAPT was extended to 16 months. However, a significant remnant was observable at 15 months follow-up.

Significant reduction of the residually perfused portion of the aneurysm sac (OKM B1–B3) was evaluated in 10 aneurysms (22%). Five of the latter (10%) revealed a small remnant (OKM B1–B3), indicating significant but yet incomplete neo-intimalization. The remaining five aneurysms showed prolonged intra-aneurysmal opacification representing significantly reduced influx accompanied by stagnation of intraaneurysmal blood (OKM A2–A3). Only one aneurysm (2%), which was located at the MCA bifurcation, demonstrated an unaltered aneurysm influx (OKM A1). Figure 4 provides an illustration of the particular case.

**Figure 5:**
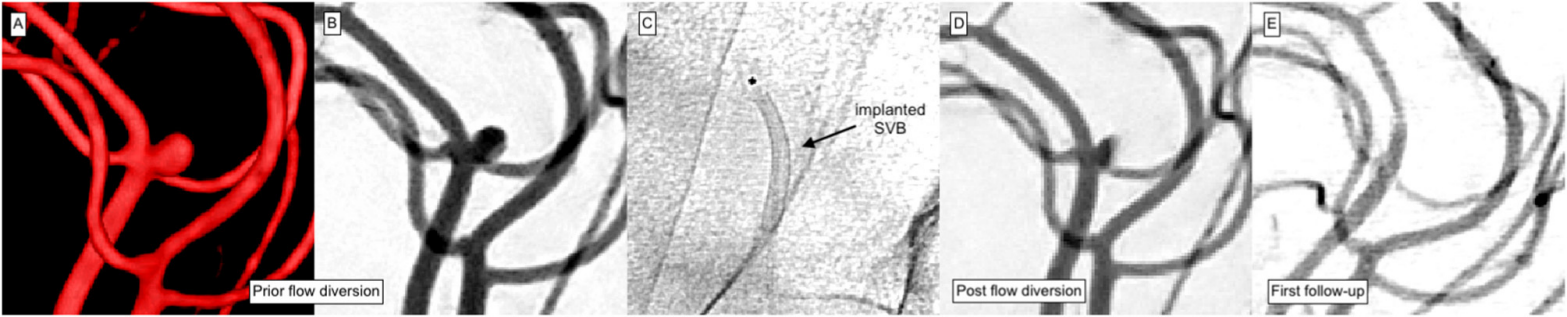
Treatment of a peripheral left ACA aneurysm. The patient exhibited further aneurysms at the contralateral MCA bifurcation and the right SUCA. **(A)** 3D angiogram demonstrates an incidental peripherally located aneurysm of the left ACA. Interdisciplinary consent for treatment using the SVB was made. **(B)** The working projection was used to quantify the target lesion and the parent artery. The saccular aneurysm measured 2.3 mm × 2.6 mm. **(C)** The SVB was implanted using the Excelsior SL10 microcatheter (Stryker Neurovascular) for device delivery. SVB was positioned within the pericallosal artery; the aneurysm arising from the callosomarginal orifice was used to center the device. **(D)** Control injection revealed immediate reduction of aneurysmal perfusion. **(E)** The first follow-up after 5 months showed the exclusion of the aneurysm from the intracranial circulation. The covered branch remains patent but exhibits a slightly reduced diameter without delayed flow.

In summary, successful treatment was observed in 98% of treatments after approximately 8 months. Figure 5 provides an example of a successfully treated distal ACA aneurysm with complete occlusion 5 months after SVB implantation. Two percent remained morphologically unaltered.

In conclusion, 45 aneurysms were available for angiographic follow-up imaging. Occlusion or significant reduction in aneurysmal perfusion (OKM: D1, C1–C3, B1–B3, and A2–A3) were observed in 44 aneurysms (98% of all aneurysms) after approximately 8 months, while the patients remained under dual platelet inhibition. Only 2% of the aneurysms remained morphologically unaltered and did not show an apparent change in perfusion (OKM A1).

## Discussion

To our best knowledge, this study is the first report on intermediate follow-up results after aneurysm treatment with the Silk Vista Baby flow diverter. Reporting efficacy, technical experiences, and adverse events that occurred in our patients is of significance, as the novel FDS is indicated and has been CE-approved for treatment of small peripheral segments of the distal intracranial arteries, a territory that is not accessible for the comparatively large flow diverters of earlier generations and thus has been applied in flow diversion therapy only recently (10).

Efficacy of endovascular treatment with the Silk Vista Baby appears to be at least comparable to earlier reports after flow diversion in more proximal locations with occlusion rates ranging from 73.3% to 89.2% at 12 months post intervention (14–16).

A major difference between these investigations and our study is the origin of the aneurysms from distal cerebral vessels, oftentimes involving bifurcations of the peripheral anterior and posterior intracranial circulation. A previous investigation by Michelozzi and colleagues reported a mean time to occlusion for bifurcation-associated aneurysms of approximately 12 months after endovascular flow diversion using the PED (Medtronic), FRED (Microvention), and Silk (Balt Extrusion) (17). Considering the available studies and our results, aneurysm treatment with the Silk Vista Baby is equally effective in distal cerebral vessels, despite the involvement of hemodynamically complex bifurcation aneurysms.

Another factor requiring consideration in this context is the duration of dual antiplatelet treatment, an essential prerequisite for the avoidance of thrombo-embolic complications, which inevitably decelerates intra-aneurysmal thrombosis, formation of the neo-intima, and, thus, aneurysm occlusion. A general guideline for DAPT in flow diversion is lacking.

In our institution, DAPT is routinely administered for 12 months aiming to avoid FDS-associated ischemic complications, which are exemplarily caused by intimal hyperplasia and delayed device induced vasospasm, as reported earlier (12). In contrast, the aforementioned studies applied DAPT for <6 months in average, followed by ASA monotherapy. Considering the significantly greater duration of DAPT in our patients, the actual efficacy of the SVB is probably even superior to the distinct FDS in those reports, as aneurysm occlusion times and rates are comparable despite the difference in hindering platelet function medication.

Technical issues were dominated by device shortening either during or shortly after implantation (5/44 patients, 11.4%). In our experience, it seems advisable to include device shortening as a relevant technical epiphenomenon into calculation for device selection. Empirically, if the proximal landing zone equals the diameter of the device or is even larger, a proximal shortening of 50% must be expected if only half of the stent is already implanted. Therefore, it seems advisable to consider the next longer version of the device in order to avoid proximal foreshortening into the aneurysm, which may jeopardize re-catheterization and further synergistic device implantations if necessary.

In seven patients, clinically apparent adverse events occurred, and one already critically ill patient died in the aftermath of the treatment during intensive care.

Two of the seven patients experienced clinically relevant prolonged or persisting neurological deficits. According to our patient population, we consider a comparatively low rate of permanent neurologic deficits of 4.8% (2 out of 41 patients). Prior studies, in contrast, reported distinctly increasing rates of clinical adverse events with persisting neurologic disorder after flow diversion of aneurysms arising from distal segments of the anterior cerebral artery and the middle cerebral artery ranging from 10% to 13% (18, 19).

The majority of symptomatic (but predominantly transient) adverse events in our patients were the sequel to focal hypoperfusion of eloquent brain parenchyma after flow diverter implantation. The latter, especially when performed in peripheral segments, causes immediate changes in loco-regional blood flow potentially manifesting with TIA-like episodes, which cease as soon as perfusion is restored to a sufficient level. In our experience, the adjustment of local perfusion, which happens within the first days after SVB implantation, can culminate in neurologically apparent ischemia despite sufficient dual platelet inhibition, especially if long-lasting episodes of vasospasm accompanied a preceding aneurysmal SAH. An exemplary case is discussed and shown in Figure 2.

The occurrence of this distinct phenomenon is more likely, if the perfusion of the MCA-bifurcation undergoes alteration via flow diversion. Especially in cases where the flow diverter requires implantation into an inferior branch of the MCA bifurcation, the orifices of one or more superior MCA branches must inevitably be covered by the hemodynamically active implant. Then, anterograde blood flow in those covered branches supplying the MCA territory close to the ACA territory is decreased and the perfusion of the border zone is further restricted. Corresponding to the pressure drop in the border zone, which is predominantly supplied by the MCA, perfusion via the ACA and its downstream leptomeningeal collaterals increases in a compensatory manner, potentially causing flow stagnation or even flow reversal within functionally connected MCA branches. During this hemodynamic adjustment, the formation of a thrombus in the respective branch is possible and—independently—the manifestation of focal neurological symptoms is comparatively frequent.

Therefore, presence of peripheral collaterals, which may cause conflicting retrograde flow in branches distal to a FDS-treated segment, has to be evaluated critically and included into treatment planning as well as patient information.

Notably, in our study, no further events occurred up to and including the last follow-up. Most importantly, delayed aneurysm rupture as a well-known critical postprocedural complication did not occur in any of our patients (20).

Comparable studies with the SVB are lacking; however, a meta-analysis investigating complication rates of flow diversion using the Pipeline Embolization Device (Medtronic) and Silk flow diverter (Balt Extrusion) revealed peri-procedural technical complications between 6.6% and 12.2%, while mortality was reported to range between 1.2 and 4.4% (21).

Our study suffers from a number of limitations. The presented results represent only a small patient collective treated in our singular institution. We therefore suggest the prospective collection of patients treated with the SVB in different centers. Furthermore, long-term angiographic follow-up data of our cohort are yet not available and should be reported as soon as they become available.

## Conclusion

Our study demonstrates the effectiveness of the Silk Vista Baby flow diverter for aneurysm treatment in small peripheral vessel segments after 8 and 11 months. Despite our comparatively long prophylactic DAPT regimen, occlusion rates are comparable to prior studies of flow diversion in more proximal locations applying significantly shorter periods of DAPT.

However, alteration of the hemodynamic equilibrium in distal localizations demands special attention to prevent ischemic events including careful supervision of patients especially in the very early post-treatment phase and a quick and comprehensive way to react to such emerging events. Therefore, a patient's collateral status and the potential effect of inevitably covered non-aneurysmal side branches should be considered in detail prior to flow diversion.

## Data Availability Statement

The original contributions presented in the study are included in the article/supplementary material, further inquiries can be directed to the corresponding authors.

## Ethics Statement

The studies involving human participants were reviewed and approved by IRB University Hospital Leipzig. The patients/participants provided their written informed consent to participate in this study.

## Author Contributions

UQ performed the interventions. EW and MS were responsible for patient monitoring and data acquisition of the post-interventional period. JM performed image analysis. NB performed review of the clinical information. FA performed vascular analysis. CR performed interventions and post-processed imaging data. K-TH wrote and reviewed the paper. CS performed interventions and follow-up imaging and analysis. SS designed the study, wrote the paper, and performed interventions. M-SS wrote the paper, performed image analysis, and statistical review. All authors contributed to the article and approved the submitted version.

## Conflict of Interest

The authors declare that the research was conducted in the absence of any commercial or financial relationships that could be construed as a potential conflict of interest.
